# Supplementary material for: Integrative analysis of the metabolome and transcriptome provides insights into the mechanisms of amino acids and their derivatives biosynthesis in Polygonatum
Source: PLoS One. 2025 Jun 25;20(6):e0327145. doi: 10.1371/journal.pone.0327145 (PMC12193713; doi:10.1371/journal.pone.0327145)
Supplement: Attachment — Submitted filename: Response to Reviewers.docx [file pone.0327145.s008.docx]

**Reply to Editor and Reviewers’ comments**

Dear Editor,

Thank you very much for kindly providing us with the opportunity to revise our manuscript entitled "**Integrative analysis of the metabolome and transcriptome provides insights into the mechanisms of amino acids and their derivatives biosynthesis in Polygonatum**". The reviewers’ comments are all valuable and very helpful for revising and improving our paper. The revised sections are highlighted in the revision. The main corrections in the paper and the response to the editor and reviewer’s comments are as follows:

**Response to Editor and Reviewers:**

**Editor:**

1. Please check the format of manuscript and make it according to journal's guidelines.

**Response:** We sincerely thank **Editor** for the meticulous comment. We have reviewed the manuscript carefully and ensure that its format aligns with the journal's guidelines. Please let me know if there are any specific areas you would like me to pay extra attention to.

2. Please divide discussion into subheadings.

**Response:** We sincerely thank **Editor** for the meticulous comment. Following your valuable suggestion, we have carefully divided the discussion section into subheadings as requested. Please find the revised conclusion in lines 321-390 of the updated manuscript.

**Reviewer #1:**

1. All the pictures are blurred, hence, i can not correlate the findings written in ms to the figures. Therefore, authors are advised to kindly provide the high resolution figures wherever it is possible because in large dataset, some picture can not be so visible that i understand.

**Response:** We sincerely thank **Reviewer #1** for the meticulous comment. We have taken the necessary steps to enhance the clarity , quality and font size of the figures. Thank you for your valuable comments.

2. All the provided deliverables are generated bioinformatically which is not so reliable till it will not be validated by wet lab experiments. The study could be validated at least targeting few amino acids gene expression analysis.

**Response:** We sincerely thank **Reviewer #1** for the meticulous comment. In response to your valuable suggestions, we have already utilized qPCR technology to conduct expression analysis on the 10 target genes selected in the manuscript. Through experimental validation, our study has become more comprehensive and reliable. Please find the revised conclusion in lines 138-155 and 309-319 of the updated manuscript.

**Other revisions to the article:** Furthermore, after careful consideration of your valuable review comments, we have not only made revisions in accordance with your suggestions but also further enhanced the results to provide a more concise summary of the article. We believe these additional modifications contribute to improving the quality of the research and the clarity of the manuscript. For further details, please refer to lines 392-402 of the revised manuscript.
