# Supplementary material for: Integrative analysis of the metabolome and transcriptome provides insights into the mechanisms of amino acids and their derivatives biosynthesis in Polygonatum
Source: PLoS One. 2025 Jun 25;20(6):e0327145. doi: 10.1371/journal.pone.0327145 (PMC12193713; doi:10.1371/journal.pone.0327145)
Supplement: Attachment — Submitted filename: Response_to_Reviewers_auresp_2.docx [file pone.0327145.s009.docx]

**Reply to Editor and Reviewers’ comments**

Dear Editor,

Thank you very much for kindly providing us with the opportunity to revise our manuscript entitled "**Integrative analysis of the metabolome and transcriptome provides insights into the mechanisms of amino acids and their derivatives biosynthesis in *Polygonatum***". The reviewers’ comments are all valuable and very helpful for revising and improving our paper. The revised sections are highlighted in the revision. The main corrections in the paper and the response to the editor and reviewer’s comments are as follows:

**Response to Editor and Reviewers:**

**Editor:**

1. Please review your reference list to ensure that it is complete and correct. If you have cited papers that have been retracted, please include the rationale for doing so in the manuscript text, or remove these references and replace them with relevant current references. Any changes to the reference list should be mentioned in the rebuttal letter that accompanies your revised manuscript. If you need to cite a retracted article, indicate the article’s retracted status in the References list and also include a citation and full reference for the retraction notice.

**Response:** We sincerely thank **Editor** for the reminder regarding the accuracy and completeness of the reference list. We have carefully reviewed all references cited in the manuscript. None of the references have been retracted. For references originally sourced from CNKI ([2], [8], [21]), we have ensured that they are cited with complete bibliographic details. These references were retained because they provide critical local or species-specific data that are not available in English literature. All necessary updates have been made in the revised reference list.

**Reviewer #2:**

1. The results are presented descriptively and rarely involve mechanistic or functional interpretation.

**Response:** We sincerely thank **Reviewer #2** for the valuable suggestion. we have added mechanistic and functional interpretations of the key findings to enrich the discussion. These revisions can be found in the “Discussion” section, particularly under “Biosynthetic Pathways and Physiological Roles of Amino Acids.” We hope this has strengthened the biological relevance and interpretative depth of our results.

2. Why are metabolite levels higher in PzP? Is it related to genetic differences, developmental conditions, or expression of regulators?

**Response:** We sincerely thank **Reviewer #1** for the insightful question. IIn the revised manuscript, we have addressed this point in the Discussion section. We propose that the elevated metabolite levels observed in PzP may be primarily attributed to transcriptional regulation. Integration of transcriptomic and metabolomic data revealed that key biosynthetic genes were more highly expressed in PzP. In addition, co-expression network analysis identified several hub genes potentially involved in regulatory control of amino acid metabolism, which were also upregulated in PzP. These findings suggest that the metabolic differences are likely due to genetic and regulatory factors, rather than only environmental or developmental conditions.

3. Can changing tryptophan levels affect levels of hormones such as IAA?

**Response:** We sincerely thank **Reviewer #2** for the important point. We agree that tryptophan is a key precursor in the indole-3-acetic acid (IAA) biosynthesis pathway in plants. In the revised discussion section, we have highlighted this relationship and proposed that the elevated tryptophan levels observed in PzP may potentially lead to increased auxin biosynthesis, which could influence rhizome development and stress responses. Although we did not directly measure IAA in this study, this connection warrants future investigation. Please find the revised conclusion in lines 360-370 of the updated manuscript.

4. The discussion should end with a more practical conclusion. For example: could this difference in metabolites be the basis for germplasm selection or genetic modification?

**Response:** We sincerely thank **Reviewer #2** for the insightful suggestion. We have revised the final paragraph of the discussion to include a more practical conclusion, emphasizing the potential application of our findings in germplasm selection and genetic improvement. In particular, we highlight that the identified key amino acid-related metabolites and biosynthetic genes may serve as valuable targets for future breeding or metabolic engineering efforts aimed at enhancing the nutritional and medicinal qualities of Polygonatum. The revised discussion now ends with a clear statement of these implications.
